# Methodological considerations for the neurophenomenology of dreaming: commentary on Windt's “Reporting dream experience”

**DOI:** 10.3389/fnhum.2014.00317

**Published:** 2014-05-23

**Authors:** Elizaveta Solomonova, Kieran C. R. Fox, Tore Nielsen

**Affiliations:** ^1^Dream and Nightmare Laboratory, Center for Advanced Research in Sleep Medicine, Biomedical Sciences, University of MontrealMontreal, QC, Canada; ^2^Cognitive Neuroscience of Thought Lab, Department of Psychology, University of British ColumbiaVancouver, BC, Canada; ^3^Dream and Nightmare Laboratory, Center for Advanced Research in Sleep Medicine, Department of Psychiatry, University of MontrealMontreal, QC, Canada

**Keywords:** dreaming, neurophenomenology, subjective experience, first-person report, third-person perspective

Windt (2013) eloquently and compellingly presents an anti-skeptical approach to the use of subjective dream reports in empirical research (the “transparency view”). On this view, dream reports are “trustworthy sources of evidence about the occurrence and phenomenal character of experience during sleep, at least when gathered under ideal reporting conditions.” Her paper is an example of the changing tides in the cognitive neuroscience of consciousness, and is a welcome valorization of the utility of subjective reports. The long-standing distrust of verbal reports of private mental processes is gradually giving way to realization of the necessity for incorporating first-person reports into objective, third-person paradigms in mutually informing ways (Varela and Shear, 1999)—a methodology often embraced by the term “neurophenomenology” (Lutz and Thompson, 2003). But what are “ideal reporting conditions?” Taking Windt's “transparency view” as a starting point, we discuss a number of methodological considerations for neurophenomenological research on dreaming.

We agree with Windt that it is crucial for empirical dream research to establish the extent to which dream reports are “transparent” accounts of subjective experiences; indeed, such transparency is the *sine qua non* for conducting meaningful qualitative and quantitative research on dream content. However, whereas dream experiences may be disclosed *to the dreamer*—or at least appear in the dreamer's memory—in a transparent way, important individual differences exist in introspective skills and in ability to articulate the breadth or depth of experience accurately in verbal or written form (Fleming et al., 2010; Sze et al., 2010; Fox et al., 2012). We may need to ask then, on both practical and epistemological levels, whether we wish to uncover what is “typical” in dreams of a certain socio-cultural population (the “breadth” of dreaming), or what is “possible” in the dream state (the “depth” of dreaming). In light of this distinction, the “ideal conditions” for reporting dreams may well be different depending on whether the purpose of a study is to assess breadth or depth of dream experience. Accordingly, and to further integrate dream studies within the nascent neurophenomenological framework, we outline two methodological elements that support more reliable elicitation, collection and analysis of dream reports: (1) specific and rigorous laboratory conditions for dream collection; and (2) introspective training and/or solicitation of “expert” participants.

## Specific laboratory conditions

Laboratory-based dream research has been based, from its early beginnings (e.g., Dement and Kleitman, 1957), on an approach that combines physiological measurement (EEG and other markers) with subjective dream reports. This has revealed qualitative and quantitative differences in the nature of dream experiences reported after awakenings from REM sleep, NREM sleep (McNamara et al., 2010), and NREM Stage 1 sleep onset (Nielsen et al., 2005; Stenstrom et al., 2012): REM sleep has been found to possess the most vivid and immersive dreams, NREM sleep the most thought-like mentation, and Stage1 NREM sleep the briefest but nonetheless REM-like mentation (Dement and Kleitman, 1957). One major limitation of the laboratory-based study, however, is the “first-night effect,” known to change sleep architecture—especially that of REM sleep—(Agnew et al., 1966) and increase the incorporation of laboratory-related content into dreams (Schredl, 2008).

Although there exist home-based sleep monitoring devices, such as the “Nightcap” (Ajilore et al., 1995), which might appear to sidestep these issues, such tools do not yet rival the variety or precision of lab-based polysomnography. Lab-based studies allow for precise electrode placement as well as for additional physiological measures, such as heart rate, respiration, muscle tone, eye movements and others, allowing much more than a simple demarcation of sleep stages. Such additional information has been profitably correlated with, and investigated alongside, sleep EEG and subjective reports (cf. Fox et al., 2013). Additionally, lab-based studies allow examination of physiological signatures of particular interest, and collection of reports in the closest possible temporal proximity to both physiological markers and dreamed experiences.

## “Expert” participants and introspective training

Different “expert” groups are used with several neurophenomenological approaches, including studies of dreaming. One such target group consists of proficient lucid dreamers who, being able to maintain awareness of their dream states, are asked to describe specific aspects of their dream experience (Fenwick et al., 1984; Lequerica, 1996; Dresler et al., 2011). Other expert groups who have been studied to access particular features of dream formation include gymnasts for their sensitivity to vestibular experience (Sauvageau et al., 1998), and vivid/frequent dreamers for their ability to access mentation reliably and with little forgetting (Stenstrom et al., 2012), among others. Introspective training for dream reporting has not been widely used in dream studies, but there is growing interest in developing such strategies (Smith, 1986; Solomonova et al., 2008). There is also evidence that expert meditation practitioners provide more accurate, objective introspective reports than non-meditators (Sze et al., 2010; Fox et al., 2012), and many meditators embrace traditions that practice observation of the dream state similar to lucid dreaming (e.g., Gillespie, 1988; Wangyal, 1998). Training participants in dream reporting, and in more generalized methods of attuning attention to one's own mental states, such as meditation (MacLean et al., 2010), may facilitate more accurate and detailed reports of dream activity (Lutz and Thompson, 2003). Although highly-trained introspectors may not reflect the full “breadth” of dream experiences for a given population, they may prove instrumental in probing the “depth” of possible mental activity in sleep (cf. Dresler et al., 2011, 2012).

We agree with Windt that trusting the dreamer to give transparent reports is a prerequisite for detailed study of subjective experiences during sleep. But a further step in reliably assessing the breadth and depth of dreaming is to ensure the greatest possible methodological support for such reporting. Providing the “ideal” reporting conditions of the sleep laboratory in conjunction with introspective training and the selection of expert participants are two particularly promising methods. Applying appropriate combinations of physiological measurements and finely tuned phenomenological interviews may yet illuminate some of the stubbornly opaque features of oneiric production, and further clarify the intricate web of relationships that bonds sleep physiology and dream phenomenology.

### Conflict of interest statement

The authors declare that the research was conducted in the absence of any commercial or financial relationships that could be construed as a potential conflict of interest.
